# A multicenter study of factors affecting nonunion by radiographic analysis after intramedullary nailing in segmental femoral shaft fractures

**DOI:** 10.1038/s41598-023-34939-6

**Published:** 2023-05-13

**Authors:** Incheol Kook, Ki-Chul Park, Dong-Hong Kim, Oog-Jin Sohn, Kyu Tae Hwang

**Affiliations:** 1 Department of Orthopaedic Surgery, Hanyang University Hospital, 222 Wangsimni-ro, Seongdong-gu, Seoul, 04763 Republic of Korea; 2 Department of Orthopaedic Surgery, Hanyang University Guri Hospital, 153 Gyeongchun-ro, Guri-si, Gyeonggi-do Republic of Korea; 3 Department of Orthopaedic Surgery, Yeungnam University Hospital, 170 Hyeonchung-ro, Nam-gu, Daegu, Republic of Korea

**Keywords:** Medical research, Risk factors

## Abstract

The factors affecting the outcomes of segmental femoral shaft fractures are currently unknown. We evaluated the outcomes of intramedullary (IM) nail fixation and investigated factors affecting nonunion of femoral shaft segmental fractures. A total of 38 patients who underwent IM nail fixation for femoral shaft segmental fractures (AO/OTA 32C2) at three university hospitals with a minimum 1-year follow-up period were retrospectively reviewed. The patients were divided into union (n = 32) and nonunion (n = 6) groups. We analyzed smoking status, diabetes mellitus, location of the segmental fragment, segment comminution, filling of the IM nail in the medullary canal, residual gap at the fracture site, use of a cerclage wire or blocking screws as factors that may affect the surgical outcome. In the union group, the average union time was 5.4 months (4–9 months). In the nonunion group, five patients required additional surgery within an average of 7.2 months (5–10 months) postoperatively, whereas one patient remained asymptomatic and did not require further intervention. On comparing the two groups, insufficient canal filling of the IM nail (union, 25.0%; nonunion, 83.3%; *p* = 0.012) and the presence of a residual gap at the fracture site after reduction (union, 31.3%; nonunion, 83.3%; *p* = 0.027) were significantly different. In the multivariate analysis, only insufficient canal filling of the IM nail was found to be a factor affecting nonunion, with an odds ratio of 13.3 (*p* = 0.036). In this study, a relatively high nonunion rate (15.8%) was observed after IM nail fixation. Insufficient IM nail canal filling and a residual gap at the fracture site post reduction were factors affecting segmental femoral shaft fracture nonunion after IM nail fixation.

## Introduction

Femoral shaft fractures have a reported incidence of 9.9–21 per 100,000 person-year, and segmental fractures account for approximately 13% of femoral shaft fractures^1,2^. Intramedullary (IM) nail fixation is the gold standard surgical treatment of femoral shaft fractures. Previous studies have reported successful outcomes after IM nail fixation for femoral shaft fractures^3^, including highly comminuted fractures (AO/OTA classification 32C3)^4–6^. Segmental femoral shaft fractures (AO/OTA classification 32C2) have little or no comminution and are more technically difficult to reduce due to the concentration of strain at the proximal and distal ends of the segment, leading to increased operative time and delayed time to union^7,8^. Therefore, the factors affecting the outcome of segmental femoral shaft fractures (AO/OTA classification 32C2) likely differ from those affecting other types of femoral shaft fractures. Although some studies have reported delayed union times after IM nailing for segmental femoral shaft fractures^7,8^, no studies have thoroughly reported on radiographic outcomes or investigated factors affecting nonunion. Therefore, this study examined the radiographic results of IM nail fixation for segmental femoral shaft fractures and analyzed the factors associated with nonunion.

## Materials and methods

### Ethical approval

This retrospective study was performed according to the Declaration of Helsinki standards and was approved by the institutional review boards of Hanyang University Hospital, Hanyang University Guri Hospital, and Yeungnam University Hospital. This study received exemption from informed consent by the institutional review board of Hanyang University Hospital.

### Patient selection

A retrospective multi-center review of the medical records of patients who underwent IM nail fixation for segmental femoral shaft fractures between July 2010 and October 2020 was conducted. Three orthopedic surgeons, all orthopedic trauma specialists with more than ten years of experience, performed all surgeries at three different university hospitals. Patients with segmental femoral shaft fractures who completed follow-up radiographs at least one year after IM nail fixation were included. Patients with open fractures, concomitant femoral fractures beyond the shaft (AO/OTA 31 or 33 fracture), major vascular injury, less than one year follow-up, or aged ≤ 18 years old were excluded.

### Assessment of outcomes

Demographic data on age, sex, smoking, diabetes mellitus (DM), follow-up period, injury mechanism were collected. Injuries were divided into low- and high-energy according to the mechanism involved. Falls from less than 1 m in height were classified as low-energy injuries, while other injuries due to greater heights or traffic accidents were classified as high-energy injuries.

**Figure 1:**
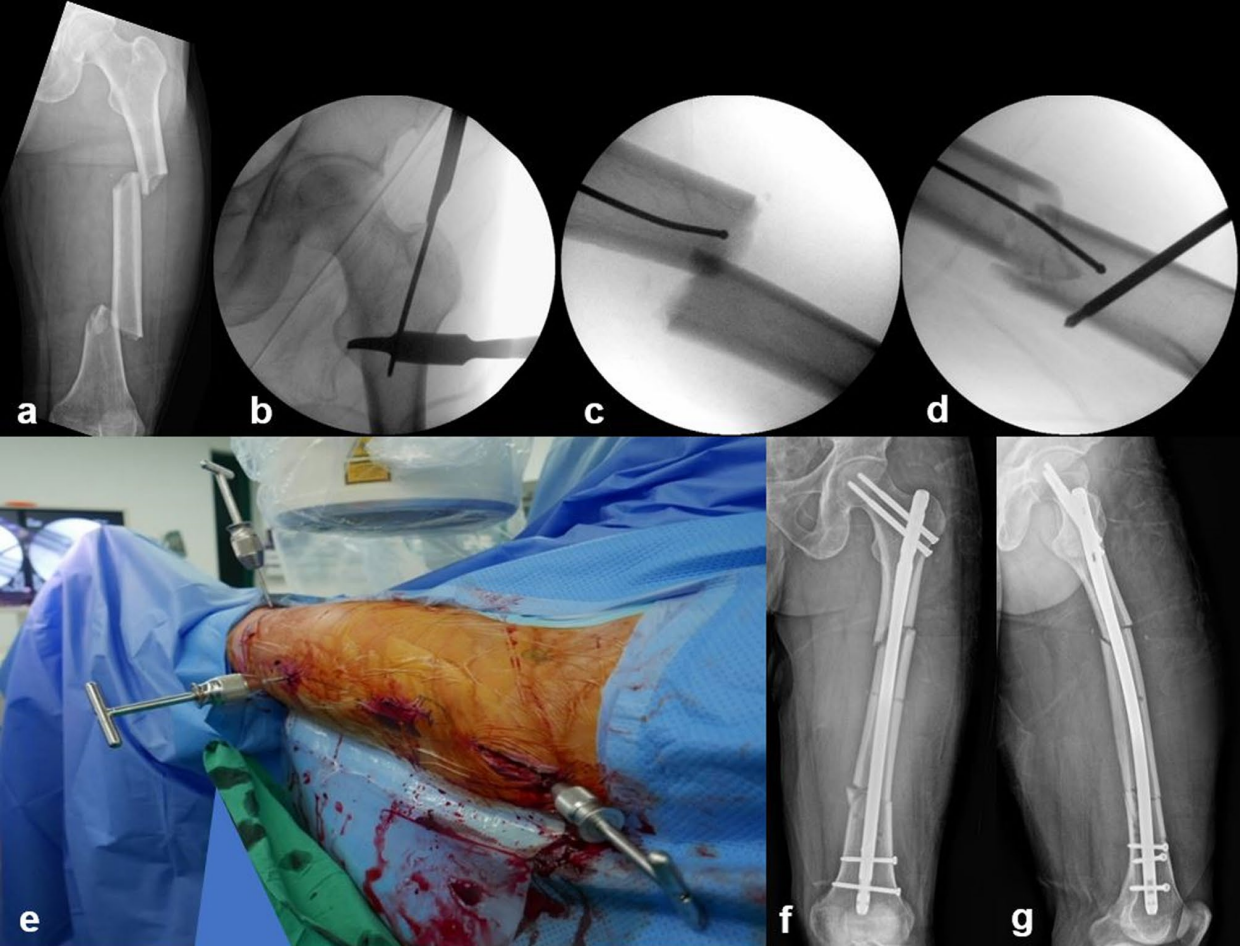
A 43-year-old male patient with a left femur segmental fracture caused by a motorcycle accident. (**a**) Plain radiograph at the initial visit. Segmental fracture was observed around the isthmus of the femur. (**b**) To aid reduction, a minimal skin incision was made in the lesser trochanter area, and the Hohmann retractor was placed on the lesser trochanter and pressed down to negate the flexion deforming force. (**c**) It was difficult to achieve reduction by a closed mean due to the absence of bony contact at the fracture site. (**d**) A percutaneous incision was made and a Schanz pin was inserted to manipulate the fracture site using the joy stick technique. (**e**) Intraoperative clinical photograph of the joystick technique. (**f**, **g**) Plain radiographs after surgery show good quality of reduction, restoring alignment, and the length of femur.

Radiographic data on the location of the fractured segment, segment comminution, surgical reduction technique, implant type, and time to union were analyzed. The fractured segment was classified by the involved anatomic area: subtrochanteric, subtrochanteric-isthmus, isthmus, isthmus-distal 1/3, and distal 1/3-supracondylar. All patients underwent surgery in a closed reduction manner. Depending on the surgeon’s decision, some cases involved minimal invasive percutaneous reduction techniques such as applying downward pressure on the lesser trochanter with long forceps or a Hohmann retractor, manipulating the segment using a Schanz pin (joy stick technique) (Fig. 1), applying a cerclage wire using cerclage wire passer, or using a blocking screw^9^. Although reamed IM nails with interlocking screws were used in all patients, the type of IM nail implant was determined by fracture location or surgeon preference. Antegrade IM nails were used in most cases with retrograde IM nails only used in four cases. Since our aim in this study was to analyze overall radiographic union after IM nail fixation, both antegrade and retrograde IM nail cases were included since both would lead to similar indirect bone healing at the fracture site.

Patients were followed up at one, two, three, six, nine months, and one year postoperatively, followed by every 6 months thereafter. Plain radiographs were taken at each follow-up visit. If delayed union or nonunion was suspected at any visit, monthly follow-up was done. Evidence of radiographic union included the presence of adequate callus bridging at the fracture site, the disappearance of the fracture line in a minimum of three cortices on anteroposterior and lateral plain femur radiographs, and union of both the proximal and distal ends of the fractured segment. The absence of an adequate bridging callus on either end of the fractured segment was not considered as union. Nonunion was defined as failure to achieve union on plain radiographs by nine months postoperatively or no visible progression of bone healing on serial plain radiographs for more than three consecutive months^10,11^.

**Figure 2:**
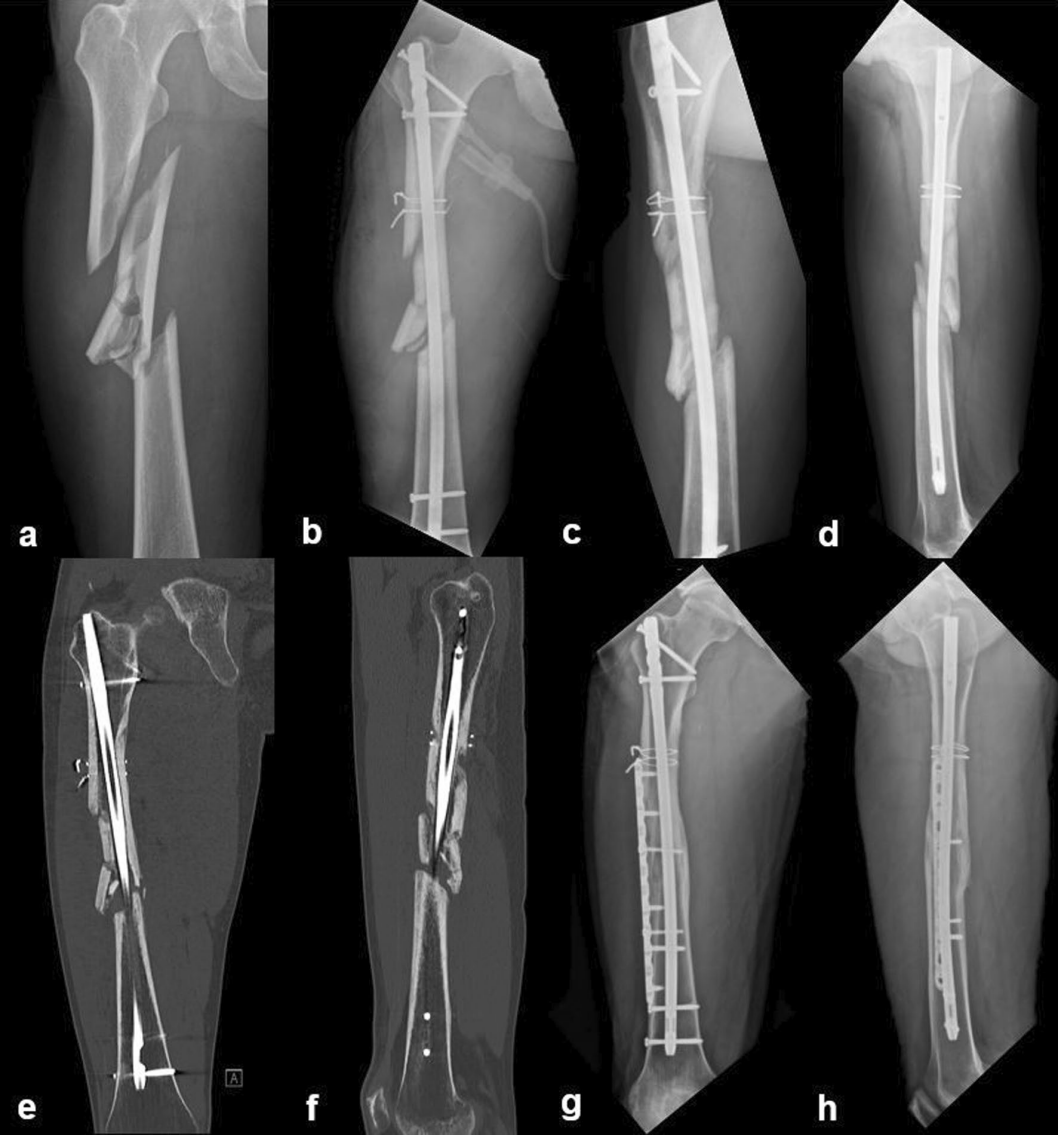
A 24-year-old male patient with a right femur segmental fracture caused by a motorcycle accident. (**a**) Plain radiograph at the initial visit. Segmental fracture was observed between the subtrochanteric area and the isthmus of the femur. (**b**) Fracture was treated with intramedullary nailing. Traction force was applied and cerclage wires were used for reduction. (**c**, **d**) Plain radiographs 9 months after surgery. Oligotrophic nonunion with little callus formation at distal isthmic area was observed. Second operation with plate augmentation was planned for nonunion. (**e**, **f**) Before the second operation, computed tomography (CT) was performed to confirm the location and extent of the nonunion. On CT coronal and sagittal planes, it is noted that there is no callus formation in the distal isthmic area. (**g**, **h**) Plain radiographs 46 months after second operation. Complete union was achieved.

Patients diagnosed with nonunion underwent computed tomography (CT) to confirm the location and extent of the nonunion prior to a second operation (Fig. 2). Nonunion was classified as hypertrophic, oligotrophic, or atrophic according to the Weber and Cech classification system^12^. A second operation was performed only when there was no progression of union on serial radiographs, or when it was deemed that union could not be achieved without additional surgery. The specific surgical approach used was based on the type of nonunion and the surgeon’s preferred method.

In order to determine the factors associated with non-union, we analyzed smoking status, DM, fractured segment location, segment comminution, filling of the IM nail in the medullary canal, residual gap at the fracture site, and use of a cerclage wire or blocking screws. For the filling of the IM nail, the distance between the inner cortex and the IM nail was measured if a gap between the IM nail and the inner cortex was visible in the isthmic portion on the immediate postoperative anteroposterior plain radiograph. In case the inner cortex and IM nail were in contact on one side, the distance was measured on the opposite side, and if both sides were out of contact, each distance was measured and summated. It was defined as insufficient filling if the measured distance was ≥ 2 mm^13^. A residual gap at the fracture site was defined as a gap of ≥ 2 mm at each end of segment on the immediate postoperative anteroposterior or lateral plain radiograph^14^. Two orthopedic surgeons who did not participate in the operations evaluated each radiograph twice, one month apart. If there was initial disagreement, the surgeons reached a consensus through discussion.

Active range of motion exercises were started immediately after surgery. Partial weight bearing was gradually increased as tolerated by the patient. The degree and timing of partial weight bearing were determined according to the patient's condition^15^. Walking aids were removed and full weight bearing was allowed when a patient was deemed to be able to walk independently. There were no significant differences in postoperative rehabilitation protocols between the involved institutions.

### Statistical analysis

An independent *t*-test was performed for continuous variables as a comparative analysis between the union and nonunion groups. The Mann–Whitney U test was used as a non-parametric test for continuous variables. For categorical variables, Fisher's exact test and chi-square test were used. To identify risk factors for nonunion, univariate and multivariate logistic regression analyses were performed using factors identified as significant among the related factors. Cohen’s kappa coefficient of agreement was used to assess the intra-observer reliability and inter-observer agreement of radiographic measurements. Statistical significance was set at a *p*-value of 0.05. Statistical analyses were performed using SPSS Statistics (version 20.0; SPSS Inc., Chicago, IL, USA).

## Results

Of the 55 patients initially identified, 17 were excluded (four due to open fractures, five due to concomitant femoral fractures, two due to vascular injury, and six due to insufficient follow-up). The final included study population consisted of 38 femurs from 38 patients. The mean patient age was 45.4 (18–74) years, and 33 patients (86.8%) were male whereas five (13.2%) were female. There were six smokers (15.8%) and four diabetic patients (10.5%). The mean follow-up period was 28.6 (12–68) months. Low-energy injuries occurred in three cases and high-energy injuries occurred in the remaining 35. All three low-energy injuries were ground-level falls whereas high-energy injuries included motorcycle accidents (16 cases), automobile accidents (11 cases), falls from height above 1 m (six cases), ski injury (one case), and crushing injury by heavy objects (one case). By segmental fracture location, one case involved the subtrochanteric area, 16 involved the subtrochanteric-isthmus area, 14 involved the isthmic area, three involved the isthmus-distal 1/3 area, and four involved the distal 1/3-supracondylar area (Table 1). Segment comminution was observed in 22 cases (57.9%). Among the patients undergoing minimally invasive percutaneous reduction techniques, 12 (31.6%) underwent cerclage wiring and two (5.3%) involved use of blocking screws. Expert Asian Femoral Nail (A2FN; Synthes, Solothurn, Switzerland) was used in 17 cases, Proximal Femoral Nail Antirotation (PFNA; Synthes, Solothurn, Switzerland) in 10 cases, Zimmer Natural Nail (ZNN; Zimmer, Warsaw, IN, USA) in seven cases and Distal Femoral Nail (DFN; Synthes, Solothurn, Switzerland) in four cases. Each IM nail was used without any version changes throughout the study. Union was obtained without additional surgery in 32 cases (84.2%), and the average time to union was 5.4 months (3–9 months). Insufficient filling of the IM nail was observed in 13 cases (34.2%), and a residual gap was found in 15 cases (39.5%) (Table 1).

**Table 1:** Demographics and surgical data of patients.

	Total (n = 38)	Union (n = 32)	Nonunion (n = 6)	p-value
Age (Years)^a^	45.4 (18–74)	45.2 (18–74)	46.7 (24–74)	0.823
Sex (M:F)	33:5	28:4	5:1	0.782
Smoking (Yes:No)	32:6	17:15	2:4	0.374
DM (Yes:No)	34:4	29:3	5:1	0.593
Follow-up period (Months)^a^	28.6 (12–68)	27.6 (12–54)	34.2 (18–68)	0.311
Injury mechanism (High)	35 (92.1%)	29 (90.6%)	6 (100.0%)	1.000
Location of segmental fragment				0.579
Subtrochanteric	1 (2.6%)	1 (3.1%)	–	
Subtrochanteric-isthmus	16 (42.1%)	14 (43.8%)	2 (33.3%)	
Isthmus	14 (36.8%)	10 (32.3%)	4 (66.7%)	
Isthmus-distal 1/3	4 (10.5%)	4 (12.5%)	–	
Distal 1/3-supracondylar	3 (7.9%)	3 (9.4%)	–	
Segment comminution	22 (57.9%)	19 (59.4%)	3 (50.0%)	0.682
Cerclage wiring	12 (31.6%)	11 (34.4%)	1 (16.7%)	0.643
Blocking screw	2 (5.3%)	2 (6.3%)	0 (0.0%)	1.000
Implant				0.714
A2FN	17 (44.7%)	13 (40.6%)	4 (66.7%)	
PFNA	6 (15.8%)	6 (18.8%)	–	
PFNA long	4 (10.5%)	4 (12.5%)	–	
ZNN	7 (18.4%)	6 (18.8%)	1 (16.7%)	
DFN	4 (10.5%)	3 (9.4%)	1 (16.7%)	
Time to union (Months)^a^	5.4 (3–9)	5.4 (3–9)	–	–
Insufficient filling of the medullary canal	13 (34.2%)	8 (25.0%)	5 (83.3%)	**0.012**
Residual gap	15 (39.5%)	10 (31.3%)	5 (83.3%)	**0.027**

Significant values are shown in bold.

DM, Diabetes mellitus; A2FN, Expert Asian Femoral Nail (Synthes, Solothurn, Switzerland); PFNA, Proximal Femoral Nail Antirotation (Synthes, Solothurn, Switzerland); ZNN, Zimmer natural nail (Zimmer, Warsaw, IN, USA); DFN, Distal Femoral Nail (Synthes, Solothurn, Switzerland).

^a^The values are given as the mean (range).

Nonunion was confirmed in 6/38 cases (15.8%), all resulting from high-energy injuries (Table 2). Regarding the type of nonunion, hypertrophic nonunion was observed in one case and oligotrophic nonunion in five cases. In four nonunion cases, the segmental fragment was located at the isthmus, and nonunion occurred at the proximal segment end. In the other two nonunion cases, the segmental fracture was located at subtrochanteric-isthmic region and the nonunion occurred in the distal segment end (Fig. 2). There was no significant difference in the nonunion rate according to surgeon (*p* = 0.760). Among the six nonunion cases, a cerclage wire was used initially in one case. Five of the six non-union cases required a second surgery, with the time to second surgery averaging 7.2 months from the initial operation. Four patients underwent plate augmentation and one underwent exchange nailing (Table 2). One nonunion patient was asymptomatic, and was followed up without additional surgery.

**Table 2:** Patients data of nonunion cases.

Sex	Age	DM	Smoking	Trauma	Injury mechanism	Fracture location	Comminution	Insufficient filling of the medullary canal	Residual gap	No. of cerclage wire	No. of blocking screw	No. of proximal locking screw	No. of distal locking screw	Type of nonunion	Implant	Second operation
M	28	**−**	** + **	MTA	High	Isthmus	**−**	** + **	** + **	0	0	2	2	Oligo	DFN	Plate augmentation
M	48	**−**	**−**	MTA	High	Isthmus	**−**	** + **	** + **	0	0	2	2	Hyper	A2FN	Exchange larger nail
M	57	** + **	** + **	In car TA	High	Isthmus	** + **	** + **	** + **	0	0	0	2	Oligo	A2FN	Plate augmentation
M	38	**−**	** + **	MTA	High	Isthmus	** + **	** + **	** + **	0	0	2	2	Oligo	A2FN	Plate augmentation
M	24	**−**	** + **	MTA	High	Subtro- Isthmus	**−**	** + **	**−**	2	0	2	2	Oligo	A2FN	Plate augmentation
M	74	**−**	**−**	Crushing injury	High	Subtro- Isthmus	** + **	**−**	** + **	0	0	2	2	Oligo	ZNN	Observation

DM, Diabetes mellitus; MTA, Motorcycle Traffic Accidents; Subtro-, Subtrochanteric area; Oligo, Oligotrophic nonunion; Hyper, Hypertrophic nonunion; DFN, Distal femoral nail (Synthes, Solothurn, Switzerland); A2FN, Expert Asian Femoral Nail (Synthes, Solothurn, Switzerland); ZNN, Zimmer natural nail (Zimmer, Warsaw, IN, USA); MIPO, Minimally invasive plate osteosynthesis.

There were no statistically significant differences between the two groups in terms of age, sex, smoking status, DM, injury mechanism, location of the segmental fragment, segment comminution, usage of cerclage wire or blocking screw, or the type of IM nail used (*p* > 0.05) (Table 1).

Insufficient IM canal filling occurred in five out of six nonunion cases, but in only eight out of 32 (25%) union cases (*p* = 0.012) (Table 1). Similarly, a residual gap of the fracture segment was observed in five of six nonunion cases (83.3%), but in only 10 of 32 (31.3%) in the union group (*p* = 0.027) (Table 1).

The odds ratio of insufficient filling of the medullary canal and residual gap at the fracture site was 14.9 (*p* = 0.021) and 11.0 (*p* = 0.039), respectively, in univariate analysis (Table 3). In multivariate analysis, insufficient filling of the medullary canal was found to be a factor associated with nonunion, with an odds ratio of 13.3 (*p* = 0.036) (Table 3). The Cohen's kappa (κ) coefficients for intra-observer reliability and inter-observer agreement by location of segmental fracture were 0.924 and 0.907, respectively. For insufficient filling, the intra-observer reliability and inter-observer agreement for both insufficient canal filling (0.917 and 0.842, respectively) and the presence of a residual gap (0.865 and 0.825, respectively) were graded as excellent.

**Table 3:** Logistic regression analysis of nonunion risk factors of femoral shaft segmental fracture.

Variables	Univariable		Multivariable	
	Odds ratio (95% CI)	p-value	Odds ratio (95% CI)	p-value
Insufficient filling	14.9 (1.52–148.32)	**0.021**	13.3 (1.19–149.99)	**0.036**
Residual gap	11.0 (1.13–106.84)	**0.039**	9.6 (0.84–110.77)	0.069

Significant values are shown in bold.

## Discussion

This study investigated the outcomes of IM nail fixation for segmental femoral shaft fractures (AO/OTA 32C2) and identified factors affecting nonunion. In our study, the overall union rate was 84.2%, lower than the previously reported 92–97.8% union after IM nailing for femoral shaft fractures^16,17^. One possibility for this discrepancy is that segmental fractures occur with higher energy injuries compared to simple or wedged femur shaft fractures and are often accompanied by more significant soft tissue damage^18,19^. Greater degrees of soft tissue damage correlate with reduced local blood flow to the injured area and poorer fracture healing, leading to a greater chance of nonunion or delayed union^20^.

In this study, six cases (15.8%) of nonunion were identified, and five (13.2%) of these nonunion cases were of the oligotrophic nonunion type. Insufficient canal filling of IM nail and the presence of a residual gap at the fracture site were identified as factors affecting nonunion. In previous reports of femoral shaft fractures, the most common nonunion type after IM nailing was hypertrophic^21^. However, we found oligotrophic non-union to be the most common non-union type in our femoral shaft segmental fracture populations. A possible reason for this difference is that segmental fractures occur in higher-energy injuries leading to a greater chance of both insufficient IM canal filling and persistence of a segmental fracture gap.

In femoral shaft segmental fractures, the segmental fragment can move or rotate relatively freely as there are no fixed parts at the fracture site and it is often out of anatomical alignment. As a result, it is difficult to achieve appropriate reduction because proper contact is required at both ends of the segmental fragment for optimal healing. Also, sufficient reaming may not be achieved since segmental fragments can move during reaming, and there is greater risk of surrounding soft tissue damage during reaming. For these reasons, it is difficult to obtain adequate reduction and perform sufficient reaming at the segmental fragment. Although open reduction would have been better for reduction quality, more stable rotational alignment, and reduced residual gap persistence, it would have exacerbated soft tissue and blood flow impairment to the fracture site already sustained by high-energy injury. This would be expected to adversely affect indirect bone healing after IM nailing and increase the complication rate. Previous studies have reported high infection rates and prolonged union times when open reduction is performed for IM nail fixation of femoral shaft fractures^22,23^. These limitations of open reduction caused our surgeons to perform minimal percutaneous reduction techniques even if there were difficulties associated with reduction and reaming.

In a retrospective case–control study of 211 patients, the authors recommended a minimum nail fit of 70% at the isthmus and ideally ≥ 90% to avoid reoperation^24^. In contrast, another retrospective study reported no correlation between nail diameter and nonunion. This lack of correlation may be due to the fact that there was only one case of insufficient IM canal filling greater than 2 mm in the study^13^. Our study included 13 cases of insufficient IM canal filling possibly due to the difficulty in reaming the segmental fragment sufficiently. Insufficient canal filling of IM nail was found to have a significant odds ratio of 14.9 and 13.4 (*p* = 0.021, 0.039) in univariate and multivariate analysis of factors affecting nonunion, respectively (Table 3). Therefore, although there is difficulty in reaming for femur shaft segmental fracture, we suggest that surgeons consider using an IM nail with the largest possible diameter.

When the IM canal is insufficiently filled, it is necessary to increase the stability of the IM nail construct. Use of a blocking screw is one way to do this. Gao et al. and Ostrum et al. reported that inserting a blocking screw inhibits unstable fixation of the IM nail, thereby facilitating fracture healing^25,26^. In our study, there were only two cases in which a blocking screw was used since the segmental fragment was mainly located in the isthmus, and both cases resulted in uneventful union.

A residual gap, observed in 10 union cases (31.3%) in the union group but only five out of six nonunion cases (83.3%), was found to be another factor affecting with nonunion. This finding is consistent with the results of previous studies in which nonunion was more common with large interfragmentary gaps in comminuted femoral shaft fractures^27–29^, and in another study which reported a fracture site gap greater than 2 mm as a risk factor for nonunion^21^. Reducing the residual gap in femur shaft segmental fractures is suggested to be an important factor for obtaining favorable outcomes^30,31^. In all of nonunion patients in our study, only one side of the segment did not unite after operation. Therefore, we suggest that the strain on the proximal and distal portion of the segment might be different. However, the number of nonunion cases was insufficient to allow for evaluating the correlation between residual gap and strain. Further studies with larger numbers of segmental femoral fractures are necessary.

In some cases, using only a closed reduction method may not achieve satisfactory reduction of the segmental fragment. Therefore, in those cases we used minimally invasive percutaneous reduction techniques. For instance, cerclage wiring was performed in 12 cases (31.6%) and it improved the reduction quality and reduced the comminuted segment fracture gap. Although no difference in union rate or infection rate has been reported in open and closed IM nail fixations^32^, extra caution is needed when performing an open reduction to minimize soft tissue injury since segmental femoral shaft fracture is a high-energy injury. Other methods, such as various percutaneous reduction techniques and forward or backward striking techniques, are thought to be more advantageous than open reductions for reducing the residual gap^30,31^.

This study has some limitations. First, this was a retrospective study with a relatively small number of patients. Femoral segmental fractures are very rare, so it was difficult to recruit a large number of patients. Larger prospective studies are needed in the future. Second, although all surgeries were performed by trauma specialists using standard operative techniques, there was the potential for confounding since three surgeons were involved and different types of implants were used. We attempted to minimize bias by enrolling patients using strict inclusion criteria. Third, this study focused on radiographic outcomes rather than other clinical outcomes such as patient function or quality of life. Since this study was conducted on patients with non-articular fractures, we assumed that most of them would have no functional problems, and the retrospective nature of this study made it difficult to assess functional status. Also, clinical evaluation was difficult because there were many cases of insufficient follow-up after fracture union. The timing and degree of postoperative weight bearing may have influenced the nonunion rate. In our study, the effect of postoperative weight bearing on nonunion was minimized because we followed a staged rehabilitation protocol that included immediate postoperative range of motion exercises and progressive partial and full weight bearing based on the patient's pain tolerance.

## Conclusions

In this study, femoral segment fractures showed a relatively high nonunion rate compared with non-segmental fractures. Factors affecting nonunion included insufficient canal filling of the IM nail and residual fracture gaps. Therefore, we suggest that use of larger diameter nails and taking steps to reduce the segment fracture gap are necessary in treatment of segmental femoral shaft fractures.

## Data Availability

The data presented in this study are available from the corresponding author on reasonable request.
